# Exposure to phages has little impact on the evolution of bacterial antibiotic resistance on drug concentration gradients

**DOI:** 10.1111/eva.12136

**Published:** 2014-01-02

**Authors:** Quan-Guo Zhang

**Affiliations:** State Key Laboratory of Earth Surface Processes and Resource Ecology and MOE Key Laboratory for Biodiversity Science and Ecological Engineering, Beijing Normal UniversityBeijing, China

**Keywords:** competitive ability, fitness costs, growth rate, mutation supply, phage therapy, resistance reversion

## Abstract

The use of phages for treating bacterial pathogens has recently been advocated as an alternative to antibiotic therapy. Here, we test a hypothesis that bacteria treated with phages may show more limited evolution of antibiotic resistance as the fitness costs of resistance to phages may add to those of antibiotic resistance, further reducing the growth performance of antibiotic-resistant bacteria. We did this by studying the evolution of phage-exposed and phage-free *Pseudomonas fluorescens* cultures on concentration gradients of single drugs, including cefotaxime, chloramphenicol, and kanamycin. During drug treatment, the level of bacterial antibiotic resistance increased through time and was not affected by the phage treatment. Exposure to phages did not cause slower growth in antibiotic-resistant bacteria, although it did so in antibiotic-susceptible bacteria. We observed significant reversion of antibiotic resistance after drug use being terminated, and the rate of reversion was not affected by the phage treatment. The results suggest that the fitness costs caused by resistance to phages are unlikely to be an important constraint on the evolution of bacterial antibiotic resistance in heterogeneous drug environments. Further studies are needed for the interaction of fitness costs of antibiotic resistance with other factors.

## Introduction

The worldwide spread of antibiotic resistance in bacterial pathogens has become a major public health problem. Factors presumed to prevent or slow the evolution of antibiotic resistance include the limitation of mutations conferring the resistance and the fitness costs associated with the resistance (Andersson 2006; Read and Huijben 2009; MacLean et al. 2010; Andersson and Hughes 2011; Cantón and Morosini 2011; Hermsen et al. 2012). However, antibiotic-resistant strains often emerge very rapidly under drug treatment, due to the typically large population sizes of bacteria and the presence of strains with intrinsic or inducible high mutation rates (Chopra et al. 2003; Henrichfreise et al. 2007; Couce and Blazquez 2009; Kohanski et al. 2010; Perron et al. 2010; Weigand and Sundin 2012). Meanwhile, it has been shown that the fitness costs of antibiotic resistance may sometimes be compensated for by additional mutations, and thus, the antibiotic-resistant strains not only spread quickly during drug treatment, but also often remain persistent or are only very slowly outcompeted by their susceptible relatives after drug use has been reduced (Schrag et al. 1997; Levin et al. 2000; Maisnier-Patin et al. 2002; Gagneux et al. 2006; Perron et al. 2010; Andersson and Hughes 2011).

In the past decade, the use of bacteriophages (phages) has received much attention as an alternative to antibiotic therapy, although bacteria may also readily evolve resistance to phages (Levin and Bull 1996; Barrow and Soothill 1997; Chanishvili et al. 2001; Summers 2001; Thiel 2004; Cairns and Payne 2008; Kutateladze and Adamia 2010; Kutter et al. 2010; Monk et al. 2010; Pirnay et al. 2011; Escobar-Páramo et al. 2012). Recently, combined use of antibiotics and phages has been shown to greatly reduce the chance of resistance evolution as there is typically little cross-resistance to phages and antibiotics (Chanishvili et al. 2001; Kutateladze and Adamia 2010; Zhang and Buckling 2012). Resistance to phages may also impose fitness costs on the bacteria, in particular when the bacteria and the phages show an evolutionary arms race in defense and counter defense (Levin and Bull 1996; Bohannan et al. 1999; Bohannan and Lenski 2000; Buckling et al. 2006; Brockhurst et al. 2007; Perron et al. 2007; Forde et al. 2008; Koskella et al. 2012). We hypothesize that treatment with phages may impact the evolution of bacterial resistance to antibiotics, as the fitness costs of resistance to phages may add to those of antibiotic resistance, further reducing the growth performance of antibiotic-resistant bacteria.

Here, we use an *in vitro* experimental system to address whether exposure to phages impacts the evolution of high-level bacterial antibiotic resistance (resistance to high-dose antibiotics) in heterogeneous drug environments. Mutations conferring resistance to low-dose antibiotics are often of high supply rates; whereas resistance to high-dose antibiotics, which is more medically relevant, may only be conferred by a combination of multiple mutations (Weinreich et al. 2006; Lozovsky et al. 2009; Read et al. 2011; Toprak et al. 2012). However, such a mutation supply limitation for high-level antibiotic resistance can be relaxed in spatially or temporally heterogeneous drug environments, where the low-dose drug environments can select for low-level antibiotic-resistant mutants that function as stepping-stones for the evolution of high-level resistance (Baquero and Negri 1997; Olofsson et al. 2005; Perron et al. 2006; Couce and Blazquez 2009; Cantón and Morosini 2011; Greulich et al. 2012; Habets and Brockhurst 2012; Hermsen et al. 2012). In such environments, fitness costs suffered by the antibiotic-resistant mutants may be an important constraint on high-level resistance evolution; and exposure to phages may reduce the level of antibiotic resistance by imposing further fitness costs (of resistance to phages) on the bacteria. In this study, we also examined the reversion of antibiotic resistance after drug use being terminated. The bacterium *Pseudomonas fluorescens* SBW25 and its lytic phage SBW25Φ2 were used as the experimental system. The bacterium and the phage can exhibit an antagonistic coevolution. On most occasions, the phage cannot drive the bacterial populations to very low densities or extinct (Vogwill et al. 2009; Escobar-Páramo et al. 2012; Zhang and Buckling 2012; Harrison et al. 2013), and thus, the phage alone does not function as a good antibacterial. However, resistance to the phage causes significant fitness costs on the bacterium (Buckling et al. 2006; Lopez-Pascua and Buckling 2008), and such fitness costs may impact the evolution of bacterial resistance to antibiotics.

## Materials and methods

### Strains and culture conditions

Two bacterial strains, *P. fluorescens* SBW25 (Rainey and Bailey 1996) and its modified variant SBW25EeZY6KX (Bailey et al. 1995), and one bacteriophage virus SBW25Φ2 (Buckling and Rainey 2002) were used in this study. SBW25EeZY6KX contains a *lacZY* insert and thus its colonies show a blue color on agar plates supplemented with X-gal, while SBW25 colonies show a yellow color. Bacteria and phages were grown at 28°C in microcosms of 200 μL of M9KB medium (M9 salt solution supplemented with 10 g L^−1^ glycerol and 20 g L^−1^ proteose peptone no. 3) in 96-well microplates. Three antibiotic drugs were use in this study: cefotaxime (CFX; Wako Pure Chemical Industries, Tokyo, Japan), chloramphenicol (CHL; Amresco, Solon, OH, USA), and kanamycin (KM; Amresco).

### Selection experiments

Figure S1 provides a schematic presentation of the experimental setup. We established 36 metapopulations, 18 with bacteria and the other 18 with both bacteria and phages. Each metapopulation consisted of ten local populations (200 μL microcosms) with a linear layout in a 96-well microplate. Each population was initially inoculated with ∼10^6^ stationary-phase bacterial cells, and, for populations with phage treatment, ∼10^4^ ancestral phage particles. For every 48 h, 2 μL (1%) of culture from each microcosm was transferred to 198 μL of fresh medium; migration within each metapopulation was then carried out by transferring 1% of each microcosm to the neighboring microcosms. The cultures were grown in antibiotic-free medium for two transfers and then were treated with single drugs, six bacteria and six bacteria/phage metapopulations for each drug. The 10 populations within each metapopulation were treated with a gradient of drug concentrations: a series of nine 2-fold dilutions (from 20480 to 80 mg L^−1^ for CFX, from 10240 to 40 mg L^−1^ for CHL, and from 2560 to 10 mg L^−1^ for KM) and a drug-free environment. The cultures were propagated for twelve transfers (∼80 bacterial generations) under drug treatment (for studying the evolution of antibiotic resistance), and then for another twelve transfers in drug-free medium (for studying the reversion of antibiotic resistance).

### Measurement of resistance level

During drug treatment, bacterial growth in each microcosm was measured as optical density (OD) at 600 nm before each transfer. We considered populations with OD ≥ 0.05 (with bacterial density > ∼10^8^ cells mL^−1^) as surviving, and populations with an OD < 0.05 as nonviable; and the level of drug resistance of a metapopulation was defined as the highest drug concentration on which OD is ≥ 0.05. At the end of drug treatment, the level of resistance for metapopulations under CFX treatment varied from 2560 to 10240 mg L^−1^; for those under CHL, from 640 to 1280 mg L^−1^, and for those under KM, from 40 to 320 mg L^−1^.

### Measurement of resistance reversion

During the twelve transfers of resistance reversion experiment, we chose one population from each metapopulation, and measure its minimum inhibitory concentration (MIC) at a 4-transfer interval. The populations to assay were from microcosms previously treated with 2560 mg L^−1^ of CFX, 640 mg L^−1^ of CHL, or 40 mg L^−1^ of KM. Before MIC assays, phages were removed from cultures: 2 μL of culture from each microcosm were transferred to 198 μL of fresh medium with 0.575% Virkon (a commercially available disinfectant; Antec International, Sudbury, England), and left static for 48 h (this procedure left the bacteria viable and completely phage free), of which 2 μL was added to 198 fresh medium and grown for 48 h to give a phage-free and Virkon-free culture (with minor modifications from Morgan et al. 2005). All cultures to assay (whether or not exposed to phages) were treated by Virkon, to minimize its influence on the measurement of MIC of different populations. A series of √2-fold dilutions of drug-containing medium (from 5120√2 to 80 mg L^−1^ for CFX, from 1280√2 to 20√2 mg L^−1^ for CHL, and from 80√2 to 5√2 mg L^−1^ for KM), together with a drug-free control, were used as the assay environments. About 2 μL of each culture was inoculated into 198 μL of each assay environment and grown for 24 h. MIC was recorded as the lowest drug concentration on which bacterial growth was blocked (OD < 0.05).

### Fitness assays

At the end of drug treatment, we sampled two populations from each metapopulation: one from the drug-free microcosm, referred to as ‘drug-susceptible’ culture, and the other from microcosms of certain drug concentrations (2560 mg L^−1^ of CFX, 640 mg L^−1^ of CHL, or 40 mg L^−1^ of KM), referred to as ‘drug-resistant’ culture. Note that due to bacterial migration during the selection experiment the ‘drug-susceptible’ cultures actually contained a low frequency of drug-resistant bacterial cells, and the ‘drug-resistant’ cultures from a particular drug concentration should also have contained bacteria of even higher resistance levels; assays using M9KB agar plates without or with drugs (320 mg L^−1^ CFX, 40 mg L^−1^ CHL, or 14.14 mg L^−1^ KM, which are the minimum inhibitory concentrations for the ancestral strain) showed that in all cases the proportion of drug-resistant cells in the ‘drug-susceptible’ cultures were lower than 7%. For all these sampled populations, growth rate was assayed in pure cultures, and relative fitness was measured by competition experiments. Before fitness assays, phages were removed from cultures by Virkon treatment; all the chosen populations (whether or not exposed to phages) and the ancestral bacteria were treated, to minimize the influence of Virkon on the measurement of fitness of different populations.

Population growth was assayed by measuring OD (at 600 nm) of pure cultures. About 2 μL of each culture was transferred to 198 μL of fresh medium and grown in an automated microplate reader at 28°C for 48 h, with OD measured at a 20-min interval. The measurements were used to fit a logistic growth model: *N*_*t*_ = *KN*_0_e^*rt*^/(*K *+ *N*_0_(e^*rt*^−1)), Where *N*_*t*_ is the observed population size (OD value) at time *t*, and three parameters to be estimated are: *N*_0_, the initial population size; *K*, the growth yield (i.e. carrying capacity); and *r*, the growth rate. Model fitting was iterated until the convergence criterion for the sum of squares (the relative reduction between successive residual sums of squares) <10^-8^ was met. The goodness of fit of the model was fairly high: in all cases, the coefficient of determination was >0.92. Here, we report growth rate as an absolute fitness measure, because it is the growth rate that determines whether or not a strain can survive the serial dilutions in our selection experiment, and growth rate has been increasingly used as a fitness measure in recent literature (Enne et al. 2004; Bell 2008; Ward et al. 2009; Perron et al. 2010). Growth yield data are presented in the Supporting Information (Fig. S2).

To measure the relative fitness, 1 μL of each of these cultures, together with 1 μL of *P. fluorescens* SBW25EeZY6KX, was inoculated into 198 μL of fresh medium and grown for 48 h. Initial and final densities of bacteria were measured by plating diluted cultures onto M9KB agar plates supplemented with X-gal (40 μg mL^−1^) on which SBW25 colonies show a yellow color and SBW25EeZY6KX colonies are blue. Fitness of the ancestral and evolved SBW25 (*W*) relative to SBW25EeZY6KX was calculated from the ratio of the estimated Malthusian parameter, *m *= ln(*N*_f_/*N*_0_)/(2 day), with *N*_0_ and *N*_f_ the relevant initial and final densities (Lenski et al. 1991); thus *W *= *m*_SBW25_/*m*_SBW25EeZY6KX_. We then calculated fitness of the evolved SBW25 populations relative to the ancestral SBW25 as an estimate of the selection coefficient (*S*) by subtracting *W* of the ancestral SBW25 from *W* of each of the evolved SBW25 population, that is, *S *= *W*_evovled_ – *W*_ancestor_ (Lopez-Pascua and Buckling 2008). Note that selection coefficients are typically calculated between ancestral and evolved genotypes in direct competition as (*m*_evolved_ – *m*_ancestor_)/*m*_ancestor_ (Lenski et al. 1991).

### Data analyses

We used the software R for data analyses (R Development Core Team 2011). To deal with the nonindependent sampling issue (e.g., repeated measures of resistance level on a same metapopulation, or measures of fitness of both antibiotic-susceptible and resistant cultures from a same metapopulation), we use mixed-effects models for data analyses (Crawley 2007). Resistance level data and MIC data, both of which log-transformed, were analyzed using mixed-effects ancova, with drug identity and phage exposure as categorical explanatory variables, time (transfer number) as a continuous explanatory variable, and metapopulation ID as a random factor. Growth rate data and selection coefficient data were analyzed using mixed-effects anova, with drug identity, phage exposure and drug resistance (susceptible versus resistant) as categorical explanatory variables, and metapopulation ID as a random factor.

## Results

### The evolution of antibiotic resistance

**Figure 1:**
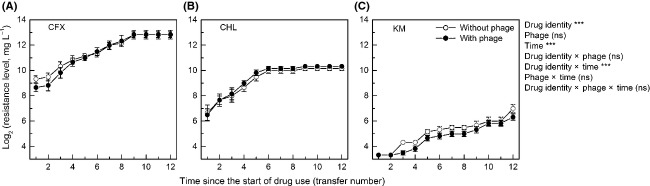
The level of antibiotic resistance in metapopulations over time during drug treatment. Data show mean ± SE. Single asterisk, *P *<* *0.05; double, *P *<* *0.01; triple, *P *<* *0.001; ns, nonsignificant.

Under drug treatment, the antibiotic resistance level increased significantly over time (time, *F*_1,390_ = 1313.72, *P *<* *0.001; Fig. 1), with the average (geometric mean) resistance level across 12 parallel metapopulations increasing from 508 mg L^−1^ to 7241 mg L^−1^ for CFX, from 95 mg L^−1^ to 1208 mg L^−1^ for CHL, and from 10 mg L^−1^ to 101 mg L^−1^ for KM. The resistance levels differed among the three drugs (drug identity, *F*_2,30_ = 592.28, *P *<* *0.001); and the trends in the increase of resistance levels over time also differed between drugs (drug identity × time, *F*_2,390_ = 10.34, *P *<* *0.001), with resistance to CFX or CHL rapidly reaching a plateau (Fig. 1A,B) and resistance to KM increasing slowly throughout the duration of drug treatment (Fig. 1C). Phage-exposed metapopulations did not differ from phage-free ones in antibiotic resistance levels (phage, *F*_1,30_ = 1.53, *P *=* *0.416), or in the pattern of increase in resistance level over time (phage × time, *F*_1,390_ = 10.34, *P *=* *0.217; drug identity × phage × time, *F*_2,390_ = 1.58, *P *=* *0.208; Fig. 1).

### The reversion of antibiotic resistance

**Figure 2:**
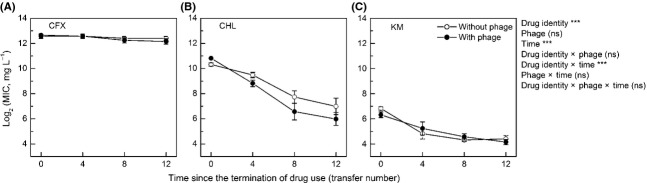
The minimum inhibitory concentration (MIC) of bacteria from microcosms of certain drug concentrations (2560 mg L^−1^ for CFX, 640 mg L^−1^ for CHL, and 40 mg L^−1^ for KM) over time since the termination of drug treatment. Symbols as in Fig. 1.

During the twelve transfers of resistance reversion experiment, the MIC of cultures previously treated with certain concentrations of drugs (2560 mg L^−1^ of CFX, 640 mg L^−1^ of CHL, or 40 mg L^−1^ of KM) decreased over time (time, *F*_1,102_ = 189.72, *P *<* *0.001; Fig. 2); with the average MIC across parallel cultures decreasing from 6267 mg L^−1^ to 4974 mg L^−1^ for CFX, from 1522 mg L^−1^ to 89.8 mg L^−1^ for CHL, and from 95.2 mg L^−1^ to 19.4 mg L^−1^ for KM. The MIC values differed among the three drugs (drug identity, *F*_2,30_ = 592.28, *P *<* *0.001), and the patterns of decrease in MIC over time also varied among drugs (drug identity × time, *F*_2,102_ = 45.67, *P *<* *0.001), with MIC under CFX decreasing much more slowly than that under CHL and KM (Fig. 2). There was not a significant difference between phage-exposed and phage-free cultures in MIC (phage, *F*_1,30_ = 2.21, *P *=* *0.147), or in the rate of changes in MIC over time (phage × time, *F*_1,102_ = 2.32, *P *=* *0.131; drug identity × phage × time, *F*_2,102_ = 2.32, *P *=* *0.103; Fig. 2).

### The fitness costs of antibiotic resistance

**Figure 3:**
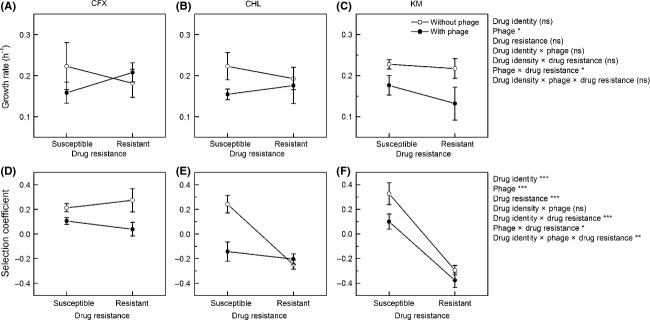
The growth rate and selection coefficient (relative fitness) of drug-susceptible and resistant bacteria sampled at the end of drug treatment. Symbols as in Fig. 1.

By doing measurements on bacterial cultures sampled at the end of drug treatment, we found that the growth rate did not show an overall difference between drug-susceptible and drug-resistant bacteria (drug resistance, *F*_1,30_ = 0.45, *P *=* *0.506), was lower in phage-exposed than in phage-free cultures (phage, *F*_1,30_ = 7.43, *P *=* *0.011); and there was a significant phage × drug resistance interaction effect: drug-resistant bacteria had lower growth rates than susceptible ones in phage-free cultures, but not in phage-exposed cultures (phage × drug resistance, *F*_1,30_ = 5.13, *P *=* *0.031; Fig. 3A–C). Cultures from different drug treatments did not differ in growth rate (drug identity, *F*_2,30_ = 0.56, *P *=* *0.579; drug identity × phage, *F*_2,30_ = 0.40, *P *=* *0.673; drug identity × drug resistance, *F*_2,30_ = 0.04, *P *=* *0.957; drug identity × phage × drug resistance, *F*_2,30_ = 0.89, *P *=* *0.420).

Overall, the selection coefficient of bacteria (difference from the ancestral strain in competitive ability) was lower in drug-resistant than in drug-susceptible cultures (drug resistance, *F*_1,30_ = 74.30, *P *<* *0.001), lower in phage-exposed than in phage-free cultures (phage, *F*_1,30_ = 17.14, *P *<* *0.001), and higher under CFX than under CHL or KM (drug identity, *F*_2,30_ = 15.47, *P *<* *0.001; Fig. 3D–F). The difference between phage-exposed and phage-free cultures in selection coefficient did not differ among drugs (drug identity × phage, *F*_2,30_ = 0.61, *P *=* *0.548); the difference between drug-susceptible and resistant cultures differed among drugs (drug identity × drug resistance, *F*_2,30_ = 23.80, *P *<* *0.001; CFX resistance did not reduce selection coefficient, but CHL-and KM resistance did so; Fig. 3) and also differed among phage-exposed and phage-free metapopulations (phage × drug resistance, *F*_1,30_ = 4.52, *P *=* *0.042; overall, this difference is smaller in phage-exposed metapopulations). Finally, there was a significant drug identity × phage × drug resistance interaction effect (*F*_2,30_ = 7.05, *P *=* *0.003): under CFX, drug-resistant bacteria had lower selection coefficient than susceptible ones in phage-exposed cultures but not in phage-free cultures (Fig. 3D), and under CHL or KM, drug-resistant bacteria had lower selection coefficient than susceptible ones, and this difference was smaller in phage-exposed than in phage-free environments (Fig. 3E,F).

## Discussion

Here, we studied the evolution of antibiotic resistance in heterogeneous drug environments. Bacterial antibiotic resistance naturally evolves in such heterogeneous environments, as low-drug habitats unavoidably exist, for example, patients with poor medication adherence in a human population, or organs and tissues of lower drug penetration within the body of a patient (Elliott et al. 1995; Mouton et al. 2008; Fantin et al. 2009). In heterogeneous drug environments, migration of bacteria from low-to high-drug habitats can facilitate the evolution of high-level antibiotic resistance, and mutation supply may not be a crucial limiting factor for the attainable level of resistance (Baquero and Negri 1997; Couce and Blazquez 2009; Greulich et al. 2012; Hermsen et al. 2012). Instead, fitness costs of resistance might be an important determinant of antibiotic resistance evolution.

### Fitness costs of bacterial resistance to antibiotics and phages, and the evolution of antibiotic resistance

The mutations conferring antibiotic resistance are often associated with fitness costs, in terms of reduced growth performance or competitive ability in the absence of the antibiotic to which they confer resistance; and such costs can be a constraint on the spread of resistant bacteria during drug treatment, and a driver of resistance reversion when drug use reduced (Andersson 2006; Read and Huijben 2009; Cantón and Morosini 2011). Resistance to phages also often causes fitness costs on the bacteria (Forde et al. 2008; Buckling and Brockhurst 2012). Here, we look at whether or not exposure to phages impacts the fitness of antibiotic-resistant bacteria. Two measures of fitness, growth rate and relative fitness (competitive ability) were investigated. The growth rate is more relevant to antibiotic resistance evolution during drug treatment as it is the growth rate that determines whether a resistant genotype can successfully spread in the presence of drug. The fitness costs of resistance to phages adding to those of antibiotic resistance may cause some antibiotic-resistant genotypes to grow very slowly, and thus retard the evolution of antibiotic resistance. The relative fitness should be more relevant to resistance reversion after drug use terminated, which is determined by to what extent the antibiotic-susceptible bacteria are competitively superior to the resistant ones. If fitness costs associated with resistance to phages augments the costs of antibiotic resistance in relative fitness (i.e., synergistic epistasis), exposure to phages may lead to faster resistance reversion after drug use reduced.

In our experiment, both resistance to drugs and resistance to phages impacted the growth rate of bacteria; however, there is an antagonistic epistasis: overall, exposure to phages caused slower growth in antibiotic-susceptible cultures, but not in antibiotic-resistant ones (Fig. 3A–C). Consistent with this, phage treatment did not show an impact on the increase of antibiotic resistance levels over time during drug treatment (Fig. 1). Relative fitness of bacteria was also impacted by the resistance. Resistance to phages showed synergistic epistasis with CFX resistance, and antagonistic epistasis with CHL or KM resistance (Fig. 3D–F). However, we observed no difference in the rate of resistance reversion between phage-exposed and phage-free cultures (Fig. 2). This might be explained by that (a) the epistatic interactions were very weak, or (b) stochasticity in migration among microcosms influenced the rate by which antibiotic-susceptible bacteria invaded microcosms of antibiotic-resistant bacteria.

An inconsistency was found between the growth rate and the relative fitness measures: it appears that both resistance to drugs and resistance to phages caused more pronounced reduction in relative fitness than in growth rate (Fig. 3). This may reflect the fact that growth rate is only one component of fitness, and the other fitness components may also be impaired by the resistance mutations. For instance, bacterial resistance to phages may result from pre-entry mechanisms such as loss of receptors for phage binding or postentry mechanisms such as degradation of foreign nucleic acids (Buckling and Brockhurst 2012), with the former likely to reduce the efficiency of nutrient uptake and thus decrease the growth rate, and the latter likely to be energy-consuming and thus decrease the growth yield of bacteria. Consistent with this explanation, we found that both resistance to drugs and resistance to phages significantly reduced bacterial growth yield (Fig. S2; bacterial growth yield data were not presented in the main text as they are less relevant to the interpretation of the resistance evolution and reversion).

We observed a difference between drugs in the increase of resistance levels over time: resistance to CFX or CHL rapidly approached to a plateau, while resistance to KM increased slowly throughout the course of drug treatment (Fig. 1). This suggests that the supply rate of mutations to KM resistance may be lower than that for CFX and CHL resistance. The speed of resistance reversion after drug used terminated also differed among drugs: CHL and KM resistance decreased more rapidly than CFX resistance (Fig. 2). This can be explained by the difference between drugs in resistance fitness costs: CFX-resistant bacteria suffered small fitness costs in relative fitness, but CHL and KM resistance caused substantial fitness reduction (Fig. 3D–F). Earlier work suggests that the fitness costs of antibiotic resistance can often be compensated for by additional mutations (Schrag et al. 1997; Levin et al. 2000; Maisnier-Patin et al. 2002; Gagneux et al. 2006; Perron et al. 2010; Andersson and Hughes 2011). This is not the case in our experiment; it is possible that the fitness costs associated with high-level antibiotic resistance cannot easily be compensated for very quickly.

### Implications for therapeutic use of antibiotics and phages

In our study, the level of antibiotic resistance was initially low and then increased over time (Fig. 1); this is consistent with previous suggestions that high-level antibiotic resistance should be less accessible than resistance to low-dose antibiotics and that migration of bacteria from low-drug habitats facilitate the evolution of resistance in high-drug habitats (Baquero and Negri 1997; Olofsson et al. 2005; Perron et al. 2006; Cantón and Morosini 2011; Greulich et al. 2012; Habets and Brockhurst 2012; Hermsen et al. 2012). Thus, it is crucial to control the rate of migration of infectious organisms from non-clinical environments to hospitals (Perron et al. 2007); meanwhile, it makes more intuitive sense to reduce migration of infectious organisms from hospitals to nonclinical environments, for preventing the spread of drug-resistant pathogens.

There was a good consistency between the relative fitness cost measures and the resistance reversion rate in our experiment: the less costly CFX resistance was lost slowly after drug use terminated, while the more costly CHL-or KM resistance declined quickly (Figs 2 and 3). This implies that measures of fitness costs of antibiotic resistance might be helpful for predicting the period of time needed for the reversion of drug resistance in clinical settings and thus may be helpful for designing drug rotational use (Brown and Nathwani 2005; Masterton 2005).

Here, we found that exposure to phages had little effect in further reducing the fitness of antibiotic-resistant bacteria. It might be worthwhile studying other factors that may impact the fitness costs of antibiotic resistance. An example is mutagenesis. Mutagenesis treatment increases the mutation loads of bacteria, and the fitness of bacteria with high mutation loads may be impaired more by further costly mutations. An earlier study with the bacterium *P. fluorescens* SBW25 and its lytic phage SBW25Φ2 (the same system as ours) showed that bacterial cultures with high mutation loads (caused by UV mutagenesis) had more rapid decline in relative fitness when coevolving with the phages during which multiple costly phage-resistance mutations should have accumulated (Buckling et al. 2006). It is possible that multiple costly antibiotic resistance mutations also had greater impact on fitness for bacteria with high mutation loads. If this proves to be true, mutagenesis treatment may help to speed the reversion of antibiotic resistance.

## Data archiving statement

Data for this study are available from the Dryad Digital Repository: http://dx.doi.org/10.5061/dryad.f9h64

## Supporting Information

**Figure S1.** Schematic presentation of the experimental set-up. **Figure S2.** The growth yield (OD600) of drug-susceptible and resistant bacteria sampled at the end of drug treatment.
