# Supplementary material for: Exposure to phages has little impact on the evolution of bacterial antibiotic resistance on drug concentration gradients
Source: Evol Appl. 2014 Jan 2;7(3):394–402. doi: 10.1111/eva.12136 (PMC3962299; doi:10.1111/eva.12136)

**Supporting Information**

Figure S1 Schematic presentation of the experimental setup. Here are shown one metapopulation with only bacteria and one with bacteria and phages. Each metapopulation consisted of ten local populations (indicated by circles) linked with 1% migration between neighbors (indicated by arrows). During the first stage, metapopulations evolved in drug-free medium for two transfers. During the second stage, cultures were treated with single drugs (cefotaxime, chloramphenicol or kanamycin) for 12 transfers; the ten populations within each metapopulation were treated with a gradient of drug concentrations (here grayscale shows drug concentration gradient). During the third stage, metapopulations evolved in drug-free medium for another 12 transfers.

Figure S2 The growth yield (OD_600_) of drug-susceptible and resistant bacteria sampled at the end of drug treatment. Data show mean ± SE. Single asterisk, *P* < 0.05; double, *P* < 0.01; triple, *P* < 0.001; ns, non-significant.


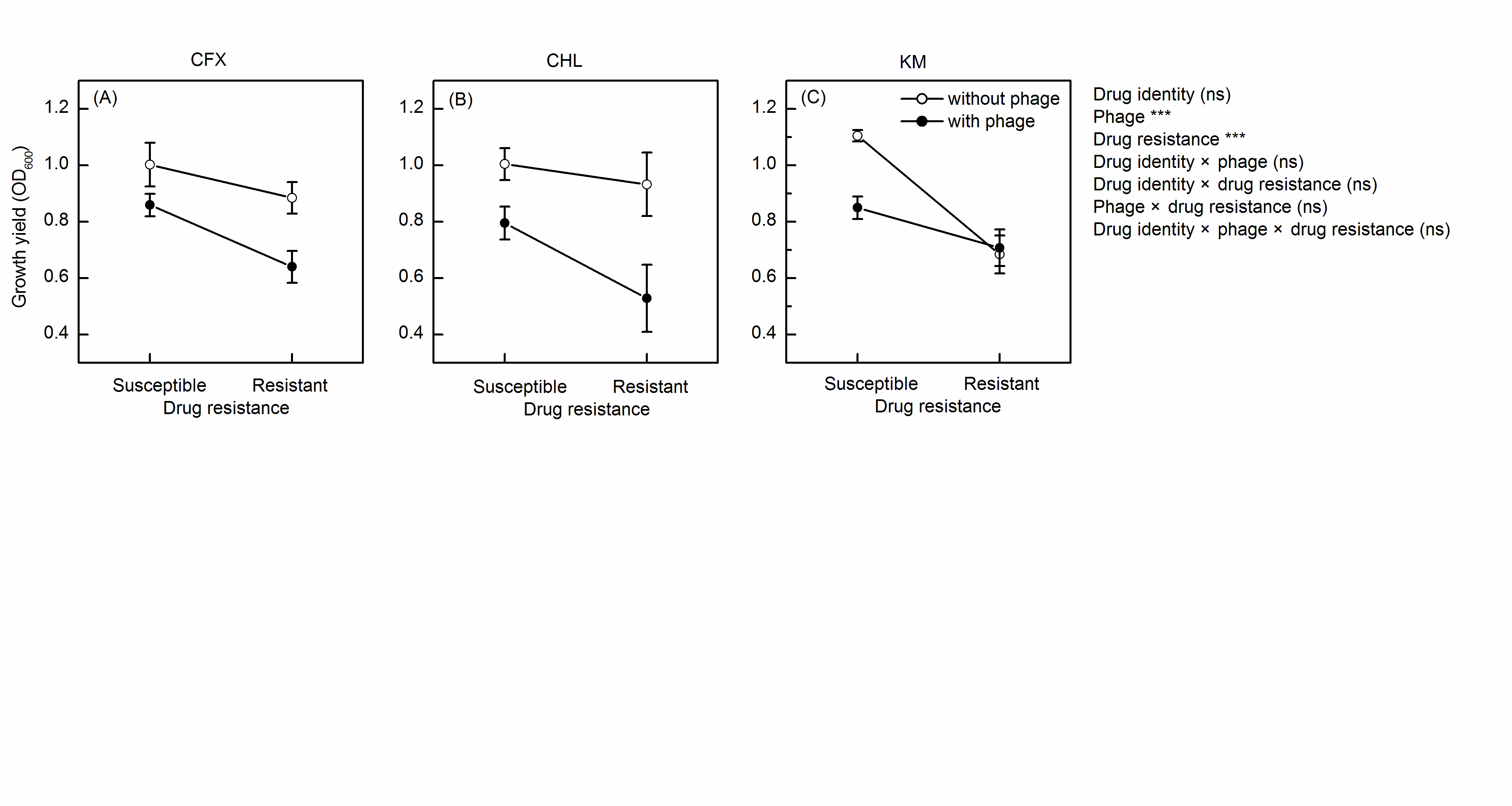

Supplement: Supplementary file 1 — Figure S1. Schematic presentation of the experimental set-up. Figure S2. The growth yield (OD600) of drug-susceptible and resistant bacteria sampled at the end of drug treatment. [file eva0007-0394-sd1.doc]
